# A Digital Self-help Intervention for Atopic Dermatitis: Analysis of Secondary Outcomes From a Feasibility Study

**DOI:** 10.2196/42360

**Published:** 2023-03-20

**Authors:** Dorian Kern, Brjánn Ljótsson, Louise Lönndahl, Erik Hedman-Lagerlöf, Maria Bradley, Nils Lindefors, Martin Kraepelien

**Affiliations:** 1 Centre for Psychiatry Research Department of Clinical Neuroscience Karolinska Institutet & Stockholm Health Care Services, Region Stockholm Stockholm Sweden; 2 Division of Psychology Department of Clinical Neuroscience Karolinska Institutet Stockholm Sweden; 3 Dermatology and Venereology Unit Department of Medicine, Solna Karolinska Institutet Stockholm Sweden

**Keywords:** atopic dermatitis, eczema, pruritus, cognitive behavioral therapy, CBT, dermatitis, skin, dermatology, self-management, self-guided, self-help, digital health, digital intervention, stress, depressive, depression, mental health

## Abstract

**Background:**

Atopic dermatitis (AD) is a common inflammatory skin disease characterized by dry skin, eczematous lesions, and an often severe pruritus. The disease may have a negative effect on quality of life and is also associated with symptoms of anxiety and depression. Few individuals with AD receive any form of behavioral intervention. Behavioral interventions for AD are potentially efficacious but need to be constructed so that they are safe, credible, and user-friendly. We have previously reported on a feasibility study that demonstrated that a self-management version of a digital intervention based on cognitive behavioral therapy (CBT) for AD can potentially be effective in reducing AD symptoms. The aim of this secondary report was to further examine treatment feasibility and preliminary effects on dermatological quality of life, itching sensations, depressive symptoms, and perceived stress.

**Objective:**

This is a secondary report on intervention credibility, usability, adverse events, and preliminary effects on secondary measures of a self-management digital intervention for atopic dermatitis.

**Methods:**

In total, 21 adults with AD, recruited nationwide in Sweden, were assessed by telephone, and used the digital intervention for 8 weeks. Participants were also assessed directly afterward and 3 months after the end of the intervention. There was no therapist guidance. Feasibility indicators included intervention credibility, usability, and possible adverse effects. Other measures included preliminary effects on dermatological quality of life, itching sensations, depressive symptoms, and perceived stress.

**Results:**

The intervention was regarded as credible and no serious adverse events were reported. System usability was, however, found to be below the predetermined cutoff for acceptable usability. Preliminary effects at 3-month follow-up were in the moderate to large range for dermatological quality of life (Cohen *d*=0.89, 95% CI 0.18-1.56), itching sensations (Cohen *d*=0.85, 95% CI 0.15-1.52), depressive symptoms (Cohen *d*=0.78, 95% CI 0.1-1.45), and perceived stress (Cohen *d*=0.75, 95% CI 0.01-1.36).

**Conclusions:**

This 8-week self-management digital CBT-based intervention was, together with telephone calls before and after, a feasible intervention for participants with AD. Preliminary effects were promising and should be explored further in a randomized controlled trial. Intervention usability was, however, rated below cutoff scores. Efforts should be made to improve written material to increase usability.

## Introduction

Up to 10% of adults have the inflammatory skin disease—atopic dermatitis (AD) [1]. AD is characterized by dry skin, rash, and itch. Treatment is aimed at decreasing inflammation in the skin and preventing symptoms. Treatment consists mainly of emollients, but when the skin is inflamed, this must be complemented with anti-inflammatory treatments, such as topical corticosteroids or calcineurin inhibitors. In more severe cases, additional treatments with UV light, systemic immunomodulators, and biological treatments are needed [1]. Extradural symptoms, such as depression and anxiety disorders are common, and psychiatric comorbidity has been found to increase in correlation with more severe eczema [2]. One study on a UK sample found that 40% of people with AD had a depression- or anxiety-related diagnosis compared to 17% of people without AD [3]. Stress and negative emotions can lead to increased scratching and skin tearing behavior. Scratching, in turn, can lead to skin damage, thus increasing inflammation—a process described as the “vicious circle of itch” [4]. Furthermore, people with AD tend to avoid situations associated with itching or other skin-related symptoms. This can lead to avoidance of important activities and, further, a decreased quality of life [5].

Cognitive behavioral therapy (CBT) for AD has been tentatively evaluated, with a focus on coping, habit reversal, and stress management, with mixed or uncertain effects [6]. We have previously developed a CBT protocol where exposure is the main treatment component. Exposure is a gradual and controlled approach to emotionally charged experiences that tend to be avoided to prevent short-term discomfort and negative emotions [7]. People with AD tend to avoid situations that they would want to experience but might trigger symptoms [5]. The purpose of exposure is to go against the initial discomfort and do what one wants in one’s life, despite the presence of symptoms or negative emotions. This exposure-based treatment was tested in a pilot study and a randomized controlled trial [8,9]. In the latter study, the treatment was delivered as a therapist-guided web-based intervention, and the results indicated moderate to large effects on eczematous symptoms and secondary measures [9]. Web-based CBT uses a similar approach to conventional CBT, usually with written feedback and guidance from a therapist [10]. However, there is a limited supply of clinical psychologists, especially those with the knowledge of AD, and to our knowledge, psychologists are not commonly found in dermatological care. An intervention without therapist support could have advantages in terms of accessibility and implementation [11]. For a self-management intervention to be as effective as its therapist-guided equivalent, it needs to be designed such that it compensates for the many potential functions of the therapist. A solid clinical process including monitoring as well as the access to well-written material and design that makes the intervention easy to use are all proposed methods to achieve this end [12]. Based on the comprehensive and therapist-guided intervention by Hedman-Lagerlöf et al [8], we developed a new self-management intervention where substantial revisions were made to adapt the treatment to a format not requiring therapist guidance [13]. The treatment material was substantially shortened, the interface streamlined, and automated messages were used to encourage participants to engage in the treatment. In a preliminary comparison, we described the differences between the 2 versions of digital CBT for AD and present some data supporting the new revised version being preliminary effective in reducing the severity of AD symptoms (69% of responders on 3-month follow-up), as well as having acceptable treatment satisfaction ratings and being associated with acceptable indicators of intervention adherence (65% of participants returned at least 5 out of 8 homework assignments) [13].

Our aim of this secondary report is to further examine the credibility, usability, and adverse events of the new self-management digital intervention for AD and present the preliminary effects on secondary measurements related to quality of life in AD and other related problems.

## Methods

### Recruitment

**Figure 1:**
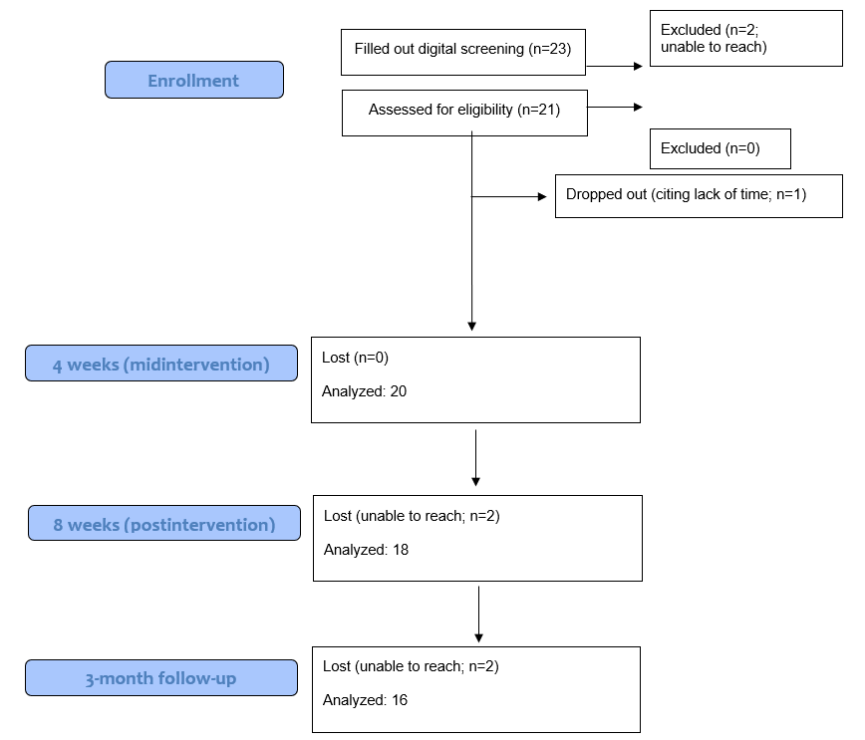
Study flowchart based on the Consolidated Standards Of Reporting Trials 2010 flow diagram.

In line with earlier similar feasibility trials [14-16] that had included 12 participants or more, we aimed to recruit at least 16 participants, to compensate for potential attrition. Eventually, 21 participants (20 female; mean age 42.5, SD 16 years) were recruited nationwide through advertisements on social media, included in the study, and started on treatment. One person dropped out, 2 were lost to postmeasurement, and 2 more were lost to 3-month follow-up. Inclusion criteria were being aged 16 years or older with a self-reported AD diagnosis. Exclusion criteria were having a disease or condition with immediate treatment priority before AD. Potential participants were screened through telephone interviews. All individuals who were interviewed were subsequently included in the study. Participants were also called after the intervention period. The purpose of the second telephone call was not to collect data, but rather for the benefit of participants who had the opportunity to summarize their experience and ask questions to the study psychologists. Recruitment started March 15, 2021, and the last 3-month follow-up assessment was collected August 20, 2021. The study flowchart is shown in Figure 1.

### Intervention

The intervention was administered on a secure website designed to resemble a mobile app. Participants read education material and used CBT-based tools. The education material starts with a short introduction to AD, and then mainly consists of how psychological and behavioral factors interact with the disease, as well as instructions on how to perform treatment exercises. The intervention content was a shortened and optimized self-management version of the therapist-guided digital CBT for AD used in the earlier described randomized controlled trial [9]. The intervention content was optimized by study psychologist DK, with the help of psychologists MK and BL. LL, a dermatologist, and NL, a psychiatrist, served as consultants. The main components were mindfulness training and exposure, same as in the original intervention, which were presented as the “Mindfulness tool” and the “Exposure tool” in the treatment [13]. These tools helped participants carry out exercises in their everyday life. A central mindfulness exercise included neutrally observing bodily sensations, such as itchiness, in a highly focused state, without attempting to judge or change any experiences. Exposure exercises are highly individualized but focus on going against unhelpful avoidance behaviors, to help participants gain more flexible behaviors. Two examples could be voluntarily putting on a woolen sweater without scratching or going to a party despite having facial eczema. The participants could gain inspiration from fictive patient examples throughout the intervention. The intervention was especially focused on exposure to an itching sensation, with scratching prevention. After performing treatment exercises, participants evaluated them with help of the program tools. Administration of the intervention was handled by DK, which included assigning treatments in the digital platform and monitoring for any automatic notices or warnings in the system—the latter indicating that participants experienced difficulties. Please see the previously reported quality improvement study for a detailed comparison of the current self-management version of the intervention, compared to the original therapist-guided version [13].

### Ethical Considerations

Study dermatologists LL and MB were consulted in the development of exercises, in order to ensure that they were safe and relevant. This study was approved by the Swedish Ethical Review Authority (2020-05702) on January 19, 2021.

### Measures

#### Overview

In addition to the measures presented below, please see Kern et al [13] for participant adherence as measured by the number of finished homework assignments, outcomes on AD severity measured with the Patient-Oriented Eczema Measure (POEM) [17], and intervention satisfaction measured with the Client satisfaction questionnaire [18].

#### Feasibility Measures

Intervention credibility was measured at the 4-week midassessment with a 5-item version of the Credibility/Expectancy Questionnaire [19]. Therein, users are asked to rate their expectations with questions such as “How successful do you think this treatment will be in reducing your symptoms?” and “How confident would you be in recommending this treatment to a friend?” The scale consists of 11-point items (0-10), yielding a score of 0-50 points in total, with higher scores reflecting higher treatment credibility. A total score of ≥30 points, reflecting the average responses to be in the upper half of the credibility ratings, is considered to indicate adequate treatment credibility [19].

Usability was measured post assessment with the System Usability Scale [20]. Therein, users are asked to rate their experience by responding to items such as “I think I would like to use this system frequently” and “I found the system unnecessarily complex.” The scale consists of ten 5-point items relating to usability, with a score range from 0-100, with higher scores reflecting better system usability and a score of ≥70 points considered to indicate acceptable usefulness [21].

Participants were also asked about any adverse effects at the end of treatment, using a self-report questionnaire where participants were asked if they have had any adverse events since the start of treatment. If they, responded with “yes,” they were asked to describe that adverse event in free text. This questionnaire is not published.

#### Outcome Measures

The Dermatology Life Quality Index [22], which measures AD-specific quality of life, ranges from 0 to 30, with a higher score indicating a lower quality of life. The authors of the scale suggest the following cutoffs: 1=no impact on quality of life, 2-5=slight impact, 6-10=moderate impact, 11-20=very large impact, and 21-30=extremely large impact.

The Peak Pruritus Numerical Rating Scale [23] measures itching sensations in the last 24 hours and uses a scale from 0=no itch to 10=worst possible itch. In this study, we only measured perceived average itch, but the scale can also be used for the worst itch during the 24 hours.

The 9-item Patient Health Questionnaire [24], measures depressive symptoms with a range from 0-27. The authors suggest cutoffs at 5 (possible depression) and 15 (probable depression).

The Perceived Stress Scale [25] measures general experience of stress using 14 items, with a range of 0-40. The authors suggest cutoffs at 0-13 (low stress), 14-26 (moderate stress), and 27-40 (high perceived stress).

Participants were asked about subjective improvement, using the Subjective Assessment Questionnaire, with a range of 0-6, using corresponding statements ranging from “much declined” to “much improved.” This type of scale is often used for other somatic conditions and has been found to be useful and valid [26].

All outcomes, collected on the web, have been frequently used in clinical practice and in several studies and have all been found to be valid and reliable.

### Statistical Analysis

Descriptive statistics were used to present observed data. Dependent samples *t* tests were performed with SPSS for Windows (version 27.0; IBM Corp). Data were analyzed per protocol. Effect sizes of within-group changes were calculated using Cohen *d* and presented with 95% CIs. Both postassessment and 3-month follow-up effect sizes were compared to the baseline preassessment effect size.

### Representative Quotes

In the final module of the intervention, participants had the opportunity to summarize and comment on their experience with this intervention in writing. To illustrate some of the general tendencies of participant feedback, 4 quotes were chosen by author DK. Two quotes were considered positive and 2 were considered negative. They were chosen to represent general tendencies, without any qualitative research method. The quotes were translated from Swedish.

## Results

### Feasibility Measures

Participants’ demographic characteristics are summarized in Table 1. Please see Table 2 for the credibility and usability of the intervention. Credibility (measured using the Credibility/Expectancy Questionnaire) was rated above the cutoff; that is, participants on average rated the intervention as acceptably credible. However, usability (measured using the System Usability Scale) was rated below the cutoff of acceptable system usability at 67 points, whereas the suggested cutoff is 70. Regarding the safety of the intervention, no serious adverse effects were reported, although some participants described a temporarily increased itching sensation in relation to exposure exercises. No participant reported a subjective deterioration of AD symptoms based on the POEM [17].

**Table 1:** Participants’ characteristics at screening (N=21).

Characteristics			Values
Age (years), mean (SD; range)			42.5 (16; 21-62)
**Gender, n (%)**			
	Males	1 (4)	
	Females	20 (96)	
**Eczema severity, n (%)**			
	Mild	3 (14)	
	Moderate	5 (24)	
	Severe	9 (43)	
	Very severe	4 (19)	
**Education level, n (%)**			
	Secondary school	6 (28)	
	University	13 (61)	
	Other	2 (11)	
Living with a partner (yes), n (%)			14 (66)
**Self-reported comorbidities (n=16), n (%^a^)**			
	Allergy	5 (20)	
	Asthma	2 (10)	
	Fatigue	1 (5)	
	Panic disorder	1 (5)	
	Atopic keratoconjunctivitis	1 (5)	
	Reactive arthritis	1 (5)	
	Endometriosis	1 (5)	
	Alopecia universalis	1 (5)	
	Glaucoma	1 (5)	
	Osteoarthritis	1 (5)	
	Thalassemia	1 (5)	

^a^Percentage of the total study population (N=21).

**Table 2:** Participant involvement, credibility, and usability of the intervention.

Measurement	Score
Returned homework assignments (n=8), mean (SD)	5.51 (2.38)
Credibility/Expectancy Questionnaire score, mean (SD; 95% CI)	33.0 (12.4; –28.66 to 94.66).
System Usability Scale score, mean (SD; 95% CI)	66.9 (16.8; –22.03 to 150.03)
Serious adverse events, n	0

### Outcome Measures

Results on outcomes and preliminary effects are summarized in Table 3. At postintervention, the outcomes showed significant improvements with small to moderate effect sizes. One exception was itching sensations, which failed to show significance. At 3-month follow-up, all outcomes were significant, with moderate to large effect sizes. On average, participants reported a score of 4, indicating a slight subjective improvement in adequate relief. Out of 18 participants at postintervention, 4 reported slight improvement, 7 reported moderate improvement, 1 person reported great improvement, and 6 reported no improvement. Participants who were not considered responders by POEM typically reported no subjective change.

**Figure 2:**
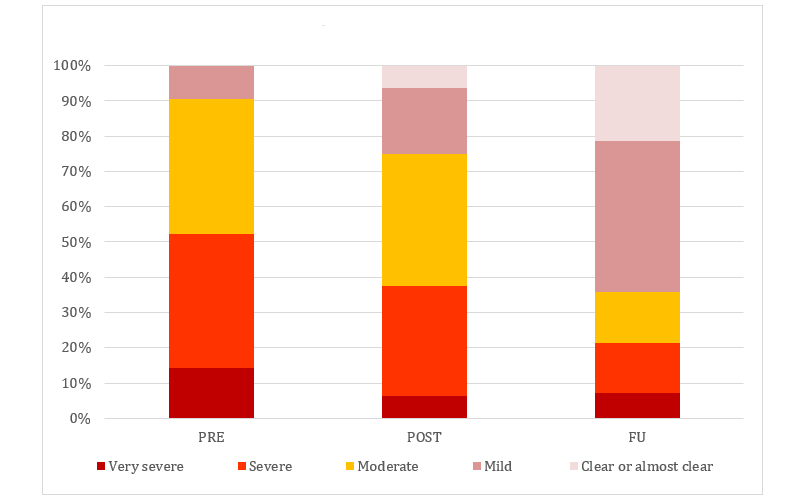
Levels of severity based on the self-rated Patient-Oriented Eczema Scale reported by Kern et al [13]. FU: 3-month follow-up; POST: postintervention; PRE: preintervention.

In Figure 2, the levels of AD severity at different assessment points are illustrated, based on the previously published outcomes on the POEM of this study [13].

**Table 3:** Outcomes and preliminary effects.

Measure		Changes from preintervention		
		Score, mean (SD)	*P* value	Effect size (95% CI)
**Dermatology Life Quality Index**				
	Preintervention	13.8 (8.3)	Reference	N/A^a^
	Postintervention	8.5 (7.9)	.049^b^	0.66 (–0.00 to 1.3)
	3-month follow-up	7 (7)	.01^b^	0.89 (0.18 to 1.56)
**Peak Pruritus Numerical Rating Scale**				
	Preintervention	5.6 (2.6)	Reference	N/A
	Postintervention	4.7 (2.7)	.28	0.36 (–0.3 to 0.98)
	3-month follow-up	3.4 (2.6)	.01^b^	0.85 (0.15 to 1.52)
**9-item Patient Health Questionnaire**				
	Preintervention	8.3 (6.0)	Reference	N/A
	Postintervention	3.8 (4.2)	.001^b^	0.87 (0.19 to 1.52)
	3-month follow-up	3.9 (5.2)	.003^b^	0.78 (0.1 to 1.45)
**Perceived Stress Scale**				
	Preintervention	18.7 (9.6)	Reference	N/A
	Postintervention	10.7 (6.9)	<.001^b^	0.91 (0.27 to 1.62)
	3-month follow-up	12.2 (9.2)	.003^b^	0.75 (0.01 to 1.36)
**Subjective Assessment Questionnaire**				
	Postintervention	4.1 (1.3)	—^c^	—

^a^N/A: not applicable.

^b^A significant change from preintervention values.

^c^Not determined.

### Participant Quotes

We present 4 quotes from participants, as shown in Textbox 1.

**Participant quotes.**

**Regarding exposure (positive):**

I am most definitely going to continue to do as I please, without letting my eczema control me […] I will continue to approach uncomfortable feelings and situations, even though it’s really hard. But it’s worth it because I am not plagued as much by the itch anymore.

**Regarding exposure (negative):**

I believe that the tool isn’t user friendly, as I find it difficult to navigate. I also feel that these exercises would do more for people with milder eczema than I have.

**Regarding mindfulness (positive):**

It is very important that I continue to practice mindfulness. Scratching is the worst trigger of eczema for me, but mindfulness helps me with the difficult task not to scratch.

**Regarding mindfulness (negative):**

It was a lot of work to fill out the mindfulness diary, but it was unclear to me what purpose it would serve. Furthermore, I thought some of the exercises were too long at 15-20 minutes.

## Discussion

### Principal Results

Our aim was to evaluate the feasibility of a web-based psychological self-management intervention for AD, based on an earlier therapist-guided and more comprehensive version. Treatment credibility was acceptable, and no serious adverse events were reported. System usability, however, was rated 3 points below the cutoff. The intervention preliminarily showed moderate to large effects on AD-related quality of life, itching, depressive symptoms, and perceived stress up to 3 months after the intervention. Overall, these results indicate that it is feasible to attempt this intervention on a larger scale. However, as system usability was, on average, rated to be below the cutoff, efforts should be made to improve system usability before carrying out a larger study. We believe that the best way to improve usability is to rework the written material, with additional and very clear instructions on how to use the tools. Based on participant feedback, participants should be better informed of the purpose of using each aspect of the mindfulness and exposure tools. However, it is also important to note that 50% of participants rated usability above the cutoff. In relation to previous research, this study supports the notion that self-guided interventions can be well-liked and useful to people with AD, if the intervention is well designed and a clinical context is provided [12].

### Limitations

There was only 1 male participant, which could imply that there is a gender-related difference in interest in this type of treatment for AD. This also implies that if there is a gender-related difference in the feasibility of this intervention, we would not have been able to find it. In addition, the effects reported are only preliminary, the sample size was small, and the intervention group was not compared to a control group. The increases in effects from postintervention to 3-month follow-up in this study is notable and could be due to external factors. Moreover, the placebo effect is assumed to be prominent in all types of interventions studies on AD and could have played a role in this study [27]. One additional external factor could be seasonal variations, as 3-month follow-up data were collected in late summer because AD symptoms are known to decrease during the summer in regions with temperate climate, such as Sweden [28].

### Comparison With Prior Work

Psychological treatment with the aim to decrease symptoms of AD is a relatively novel idea, and the results supports the use of CBT with mindfulness and exposure for AD, similar to previous studies [5,9]. In comparison to previous interventions, this intervention is characterized by its brief content, unguided and internet-delivered format, and focus on mindfulness and exposure.

### Conclusions

The results of this study showed the intervention to be acceptable, potentially effective, and safe. Considering these preliminary results, we believe that it would be feasible to conduct a randomized controlled trial, with the self-management intervention being compared directly against the therapist-guided intervention. This is conditional to the written material being improved to offer better guidance to the mindfulness and exposure tools, as the usability was below the cutoff. Future studies should attempt to recruit more male participants so that any potential gender differences in treatment preference and response could be explored. A further consideration for future research is to investigate the feasibility of this method to adapt psychological treatments for disorders other than AD.

## Abbreviations

AD: atopic dermatitis
CBT: cognitive behavioral therapy
POEM: Patient-Oriented Eczema Measure
